# FOXA2 Activates RND1 to Regulate Arachidonic Acid Metabolism Pathway and Suppress Cisplatin Resistance in Lung Squamous Cell Carcinoma

**DOI:** 10.1111/crj.13814

**Published:** 2024-08-11

**Authors:** Yafu Zhou, Huiguo Chen, Jianhua Yan, Qi Yao, Chunchu Kong, You Peng, Shengying Xiao, Jinsong Yang

**Affiliations:** ^1^ Department of Thoracic Surgeons Hunan Provincial People's Hospital (The First Affiliated Hospital of Hunan Normal University) Changsha China; ^2^ Department of Respiratory Hunan Provincial People's Hospital (The First Affiliated Hospital of Hunan Normal University) Changsha China; ^3^ Department of Geriatric Hunan Provincial People's Hospital (The First Affiliated Hospital of Hunan Normal University) Changsha China; ^4^ Department of Oncology Hunan Provincial People's Hospital (The First Affiliated Hospital of Hunan Normal University) Changsha China

**Keywords:** arachidonic acid, DDP resistance, lung squamous cell carcinoma, RND1

## Abstract

**Background:**

The primary cause of cancer‐related fatalities globally is lung cancer. Although the chemotherapy drug cisplatin (DDP) has brought certain benefits to patients, the rapid development of drug resistance has greatly hindered treatment success.

**Methods:**

We used the lung squamous cell carcinoma (LUSC) mRNA data set to explore the differentially expressed gene (RND1) in LUSC and detected RND1 expression in LUSC cells and DDP‐resistant cells by qRT‐PCR. Meanwhile, we performed abnormal expression treatment on RND1 and conducted CCK8, colony formation, and flow cytometry to evaluate the impact of RND1 expression on cell proliferation, apoptosis, and DDP resistance. In addition, we analyzed metabolism pathways involving RND1 using GSEA. We also used online tools such as hTFtarget and JASPAR to screen for the upstream transcription factor FOXA2 of RND1 and verified their relationship through CHIP and dual luciferase experiments. Finally, we validated the role of FOXA2‐RND1 in DDP resistance in LUSC through the above experiments.

**Results:**

RND1 was downregulated in LUSC, and overexpression of RND1 repressed proliferation and DDP resistance of LUSC cells and facilitated cell apoptosis. RND1 modulated the arachidonic acid (AA) metabolism pathway, and FOXA2 positively manipulated RND1 expression. By activating FOXA2, stabilizing RND1, and regulating AA levels, the sensitivity of LUSC cells to DDP could be enhanced.

**Conclusion:**

Our study suggested that FOXA2 positively modulated the RND1‐AA pathway, which repressed the resistance of LUSC cells to DDP.

## Graphical abstract

FOXA2 activates RND1 to suppress the arachidonic acid metabolism pathway and cisplatin resistance in lung squamous cell carcinoma.

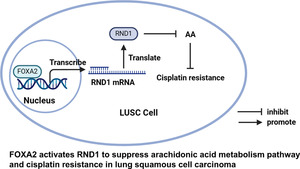

## 1 Introduction

The most prevalent malignant tumor in the world, lung cancer, has a poor survival rate and a high likelihood of recurrence after surgery, making it one of the main causes of cancer death globally [1]. A total of 20%–30% of lung cancer fatalities are caused by lung squamous cell carcinoma (LUSC), a prevalent subtype of lung cancer [2]. About 40% of LUSC patients still have a chance of recurrence during the following 5 years, even after possibly curative surgery [3]. Cisplatin (DDP)‐based systemic chemotherapy remains the standard course of action for LUSC patients [4]. It can restrain cancer cell growth by binding to tumor cell DNA and fostering cell apoptosis [5]. Generally, LUSC patients are relatively sensitive to initial chemotherapy. However, long‐term use of chemotherapy drugs can gradually lead to the development of drug resistance in tumor cells, resulting in treatment failure and a reduced patient survival rate [6]. DDP resistance is often brought on by multiple factors and mechanisms. This includes upgrades in intracellular repair mechanisms, changes in transport proteins, abnormalities in apoptosis pathways, and alterations in the tumor microenvironment [7, 8, 9]. Hence, it is urgent to identify novel molecular targets that affect the development of secondary chemoresistance in LUSC patients. An in‐depth understanding of drug resistance mechanisms and the investigation of novel strategies may enhance the sensitivity of tumors to DDP and improve therapeutic outcomes and survival rates for patients.

Enzymes and proteins involved in the metabolism pathways in vivo influence the metabolism rate, transformation products, and clearance ability of chemotherapy drugs. Tumor cells modulate metabolism pathways to affect their metabolic response to chemotherapy agents, thus affecting efficacy and drug resistance [10, 11]. In addition to well‐known pathways such as glycolysis and metabolism reprogramming, arachidonic acid (AA) has also been identified as a pivotal lipid signaling molecule. AA and its metabolites affect the growth, proliferation, and metastasis of tumor cells, and are associated with drug resistance [12]. AA can produce prostaglandins (PGs) through the action of PTGS1 (COX‐1) and/or PTGS2 (COX‐2), thereby influencing normal development, tissue homeostasis, inflammation, and cancer progression [13]. AA may serve as an apoptotic signal, modulating apoptosis, and metabolism produces PG, especially PGE2, which facilitates inflammation and cancer progression [14, 15]. Additionally, the AA pathway can activate the expression of proteins linked to lung cancer drug resistance [16]. In order to increase the effectiveness of tumor treatment, the influence of the AA metabolism pathway should be taken into account. Additionally, more research into associated molecular targets is required.

RND1 is supported to be an atypical member of the Rho family GTPase 1 [17]. The RND family is a crucial protein family, which includes RND1, and they play vital roles in cellular biological processes. Essentially, the RND family is mainly involved in manipulating cell morphology and the dynamic reorganization of the cytoskeleton. Due to this function, it is crucial for cell migration, invasion, and support of cell structure [18]. The classic imbalance of Rho GTPase activity can influence multiple pivotal physiological processes in tumor cells, such as migration, metastasis, invasion, and angiogenesis [19]. During these physiological processes, RND1 exerts a modulatory effect. RND1 is generally considered a tumor repressor, and its loss or aberrant expression in tumor cells may lead to tumor onset and development [20, 21, 22]. In breast cancer, RND1 downregulation facilitates tumor occurrence and EMT through Ras‐MAPK signaling [20]. Besides, low RND1 expression is implicated in an unfavorable prognosis in patients. In liver cancer, patients with lower levels of RND1 have larger tumor volumes and are more prone to microvascular invasion [23]. One mechanism leading to low expression of RND1 in tumors is achieved through the modulation of transcription factors. Research is still needed to identify upstream modulatory genes of RND1 in cancer induction.

In this work, the bioinformatics approach was utilized to identify RND1 as a modulator of the AA metabolism pathway, and FOXA2 was identified as an upstream regulator of RND1. The regulatory relationship between them was experimentally validated. Furthermore, experimental methods such as CCK8, colony formation, and flow cytometry were applied to investigate the molecular mechanisms involved in AA metabolism and DDP resistance in LUSC.

## 2 Materials and Methods

### 2.1 Cell Culture

Human embryonic kidney cells (293 T), human normal lung epithelial cells (BESA‐2B), LUAC cells (SK‐MES‐1), and human non–small cell lung cancer cells (H1299, A549) were all accessed from the BeNa Culture Collection (China). SK‐MES‐1/DDP, a DDP‐resistant strain, was purchased from the WHELAB company (China). 293 T and BEAS‐2B cells were maintained in DMEM‐H containing 10% fetal bovine serum (FBS) and 1% P/S. The medium for 293 T cells also contained 2 mM L‐glutamine. H1299 cells were maintained in RPMI‐1640 containing 10% FBS and 1% P/S, with A549 cells in F‐12 K containing 10% FBS and 1% P/S, and SK‐MES‐1 and SK‐MES‐1/DDP cells in MEM (with NEAA). All cells were kept in an incubator at 37°C with 5% CO_2_.

### 2.2 Cell Transfection

oe‐NC, oe‐RND1, si‐NC, and si‐FOXA2 plasmids were purchased from Genechem (China). SK‐MES‐1 or SK‐MES‐1/DDP cells were cultured to an appropriate density and plated in culture dishes. Lipofectamine 2000 reagent (Invitrogen, United States) was employed to mix an appropriate amount of Lipofectamine 2000 reagent with the corresponding plasmid to prepare the transfection mixture. The transfection mixture was dropwise added to the culture dish, and the dish was gently shaken to ensure even distribution of the mixture. Finally, culture dishes were placed back in the incubator for further cultivation.

### 2.3 RNA Extraction and qRT‐PCR

Cells were treated with TRIzol (Invitrogen, United States) to isolate total RNA. Afterward, the amount of RNA was measured with a NanoDrop ND‐1000 spectrophotometer (NanoDrop Technologies, United States). Reverse transcription was conducted to synthesize cDNA using the PrimeScript™ II 1st Strand cDNA Synthesis Kit (Takara, Japan). Subsequently, a qRT‐PCR assay was done with TBGreen® Premix ExTaq™ (Takara, Japan) on an Applied Biosystems™ 7500 real‐time PCR system. GAPDH was utilized as an internal reference. The data were calculated using the 2^−ΔΔCT^ method to obtain relative expression levels. The primer sequences used were as follows: FOXA2 (F: TGCACTCGGCTTCCAGTATG, R: CGTGTTCATGCCGTTCATCC), RND1 (F: CGCAACCCTCCCTTCTGAAT, R: TGGGCACATAGGTCTCTGGA), PTGS1 (F: AGACCACTGCTGTGCTTCTC, R: GAAACAGCTGCTCACCTACG), PTGS2 (F: TCCAGCCCCACTCCTAATGA, R: TGTCCTTTAATTGCAGCAAATCC), PLA2G4A (F: GCTAGAGGCATTGAGGAGCC, R: TGTCACTTTGGTGGCACGTA), and GAPDH (F: AATGGGCAGCCGTTAGGAAA, R: GCGCCCAATACGACCAAATC).

### 2.4 Determination of IC_50_ of DDP by CCK8

5 × 10^3^ cells were plated in each well of a 96‐well plate with 100 μL of culture medium. After the cells adhered to the wall, the supernatant was discarded, and then the cells were treated with a culture medium containing different concentrations of DDP (0, 15, 30, 45, 60, 75, and 90 μg/mL) for 24 h. Afterward, 10 μL of CCK8 detection solution (Beyotime, China) was added to each well, and the absorbance at 450 nm was assayed, and the DDP IC_50_ value was calculated after incubation for 4 h.

### 2.5 Colony Formation Experiment for Cell Proliferation Assessment

Cells in the logarithmic growth phase were digested with trypsin and resuspended in cell suspension. Cells were plated in a 6‐well culture plate (500 cells/well). Cells were maintained until the number of cells in a single colony exceeded 50, and the medium was replaced every 3 days to observe cell status. After cloning, cells were rinsed once with PBS (Solarbio, China) and fixed with 1 mL of 4% paraformaldehyde per well for 30 min, followed by another rinse with PBS. 0.5% crystal violet staining solution (1 mL) was added to each well, and the cells were stained for 30 min. Cells were subjected to several PBS rinses, air‐dried, and pictured with a digital camera.

### 2.6 Flow Cytometry Analysis of Apoptosis Rate of SK‐MES‐1/DDP Cells

Annexin V‐FITC/PI Apoptosis Kit (Multisciences [Lianke] Biotech, China) was utilized to assess apoptosis levels. In brief, cells from varying groups were plated in a 6‐well plate and cultured for 24 h (3 × 10^5^ cells/well). Following being washed three times with PBS, cells were treated with 5 μL Annexin V‐FITC or 10 μL PI and incubated at room temperature in the dark for 15 min. Cell apoptosis levels were assayed on the NovoCyte flow cytometry system (Agilent, United States), with Annexin V‐FITC as green fluorescence and PI as red fluorescence.

### 2.7 Coimmunoprecipitation (Co‐IP)

RIPA lysis buffer (Thermo Scientific, United States) was utilized to lyse 1 × 10^8^ LUSC cells on ice for 15–30 min. The supernatant was collected by high‐speed centrifugation at 4°C for 10 min after cell lysis. The supernatant was divided into an input group and an IP group, and FOXA2‐specific antibodies (ab256493, Abcam, United Kingdom) were used with IgG (ab172730, Abcam) as negative controls. Subsequently, the supernatant of each group was taken and incubated overnight with antibodies by rotating at a low temperature. After incubation, the supernatant was centrifuged for 1–2 min to collect overnight. Next, Protein Agar/Sepharose (ab193262, Abcam) was incubated with the supernatant at a low temperature for 2 h, and the precipitate was collected by centrifugation for 2 min. Subsequently, the protein on the agarose beads was eluted for western blot analysis.

### 2.8 CHIP Assay of Binding Relationship Between FOXA2 and RND1

LUSC cells were fixed with formaldehyde for 10 min and then subjected to ultracentrifugation. The supernatant was collected and divided into two tubes. One tube was incubated overnight at 4°C with IgG (ab172730, Abcam), and the other tube was incubated overnight at 4°C with FOXA2‐specific antibody (ab256493, Abcam). Protein Agar/Sepharose (ab193262, Abcam) was added to both tubes for precipitation, followed by centrifugation at 12 000 g for 5 min and removal of the supernatant. Nonspecific complexes were washed. Next, the samples were subjected to overnight decrosslinking at 65°C. qRT‐PCR was conducted using RND1‐specific primers to detect the binding of FOXA2 and RND1.

### 2.9 Dual‐Luciferase Reporter Gene Assay

To verify the binding between FOXA2 and RND1, a dual‐luciferase reporter assay was done using the Dual‐Luciferase Reporter Assay System (Promega, United States). First, PGL3‐RND1‐WT and PGL3‐RND1‐MUT plasmids were established. The plasmid was cotransfected with si‐NC or si‐FOXA2 into 293 T cells. Cells were harvested 48 h later, and luciferase activity was detected according to the instructions of the Dual‐Luciferase Reporter Assay System (Promega, United States).

### 2.10 Western Blot

Cells were plated (4 × 10^5^ cells/well) in a 6‐well plate, and transfected LUSC cells were collected and lysed with RIPA buffer (Thermo Fisher, United States) to extract total proteins. Protein concentration was assayed with a BCA assay kit (Beyotime, China). Equal amounts of protein samples were separated on SDS‐PAGE and transferred to the PVDF membrane (Thermo Fisher, United States). After blocking with 5% skim milk, the membrane was incubated with primary rabbit antibody overnight at 4°C, followed by washing and incubation with secondary antibody for 2 h. Finally, protein bands were detected using an ECL detection reagent (Millipore, United States) and a fluorescence and chemiluminescence imaging system (Clinx, China). Antibody information was as follows: FOXA2 (ab256493, Abcam, United Kingdom), RND1 (PA5–121635, Invitrogen, United States), PTGS1 (ab109025, Abcam, United Kingdom), PTGS2 (ab179800, Abcam, United Kingdom), PLA2G4A (ab307889, Abcam, United Kingdom), β‐actin (ab8227, Abcam), and goat anti‐rabbit IgG H&L (HRP) (ab6721, Abcam).

### 2.11 Bioinformatics Analysis

LUSC mRNA count data were collected from The Cancer Genome Atlas (TCGA; https://portal.gdc.cancer.gov/) database, including 49 normal tissue samples and 502 tumor tissue samples. Differential analysis was conducted with the “edgeR” package to identify genes with significant differential expression (logFC > 1.5, FDR < 0.05). Based on relevant literature, these differentially expressed genes (DEGs) were determined as target gene mRNAs. Next, the upstream potential transcription factors of the target gene were predicted using the hTFtarget (http://bioinfo.life.hust.edu.cn/hTFtarget#!/). Then, the upstream transcription factor associated with the target gene was determined using the Pearson correlation method. In addition, the motif sites of this upstream transcription factor were predicted using the JASPAR database (https://jaspar.genereg.net/). Finally, GSEA was conducted on the target gene.

### 2.12 Data Analysis

All experiments were independently repeated three times. Data were analyzed and plotted using GraphPad Prism, and the data were presented as mean ± SD. A Student's *t*‐test was utilized for two‐group comparisons, and a one‐way ANOVA was used for multiple comparisons. *p* < 0.05 is considered statistically significant.

## 3 Results

### 3.1 Low Expression of RND1 Fosters DDP Resistance in LUSC Cells

**FIGURE 1:**
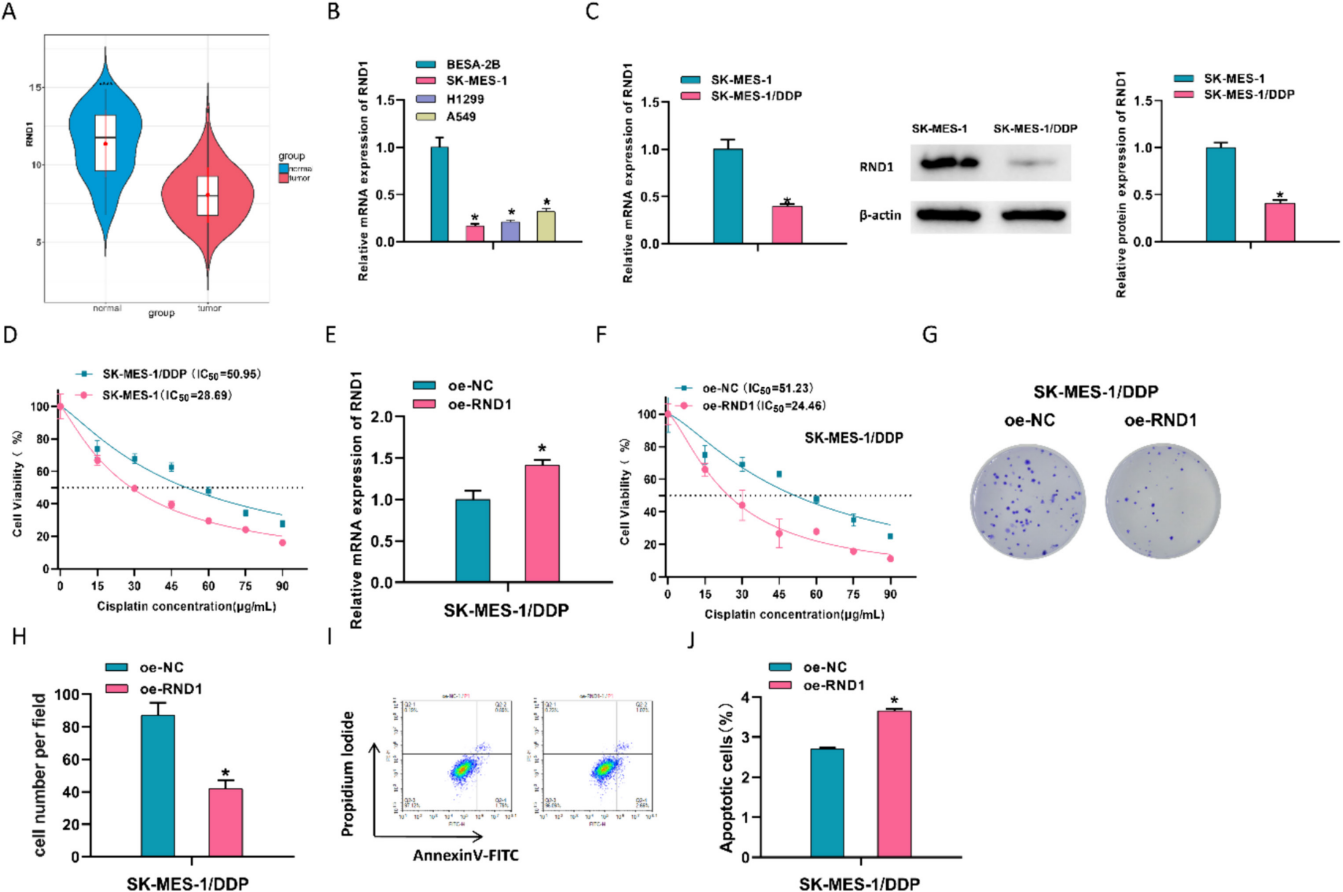
RND1 expression in LUSC and effects of abnormal expression on cancer cell DDP resistance, proliferation, and apoptosis. (A) Violin plot showing the expression levels of RND1 in LUSC tumor tissues and adjacent tissues. (B) qPCR detection of RND1 expression levels in human normal lung epithelial cells (BESA‐2B), LUSC cell line (SK‐MES‐1), and human non–small cell lung cancer cell lines (H1299 and A549). (C) qPCR and western blot detection of RND1 mRNA and protein expression levels in DDP‐resistant and sensitive SK‐MES‐1 cells. (D) CCK8 analysis of DDP IC_50_ values in SK‐MES‐1/DDP and SK‐MES‐1 cells. (E) qPCR detection of transfection efficiency of overexpressed RND1 in SK‐MES‐1/DDP cells. (F) CCK8 analysis of DDP IC_50_ values for oe‐RND1 and oe‐NC group. (G, H) Images and quantitative analysis of colony formation assay in LUSC cells transfected with oe‐NC and oe‐RND1. (I, J) Cell apoptosis rate and quantification graphs of LUSC cells transfected with oe‐RND1 as assayed by flow cytometry. * represents *p* < 0.05.

This study analyzed the mRNA data of LUSC in TCGA database. Compared with normal tissues, RND1 was downregulated in LUSC tissues (Figure 1A). As assayed by qPCR, RND1 was significantly downregulated in three cancer cell lines compared to the normal cell line (Figure 1B). Based on previous experience, we speculated that RND1 acted as a tumor repressor in LUSC. To delineate the molecular mechanisms of DDP resistance in LUSC, we purchased two cell lines, including the DDP‐resistant strain SK‐MES‐1/DDP and the sensitive strain SK‐MES‐1. RND1 levels in two types of LUSC cells were assessed by qRT‐PCR and western blot. RND1 had relatively high expression in sensitive cell lines (Figure 1C). The CCK8 experiment results showed that, compared with SK‐MES‐1 cells, SK‐MES‐1/DDP cells significantly increased the IC_50_ value of DDP (Figure 1D). Thus, we overexpressed RND1 in the resistant strain and detected transfection efficiency by qRT‐PCR. As plotted in Figure 1E compared with the NC group, RND1 in the oe‐RND1 group was significantly elevated. The IC_50_ values of DDP in different groups were determined by the CCK8 assay. Compared with the control, the IC_50_ value of the oe‐RND1 group was significantly reduced (Figure 1F). Then, we treated SK‐MES‐1/DDP cells with IC_50_ and assessed the proliferation and apoptosis of LUSC cells using colony formation and flow cytometry. Increased RND1 significantly hindered the proliferation of LUSC cells (Figure 1G,H). Meanwhile, increased RND1 significantly facilitated apoptosis of LUSC cells (Figure 1I,J).

### 3.2 RND1 Modulates AA Metabolism and Thus Affects LUSC DDP Resistance

**FIGURE 2:**
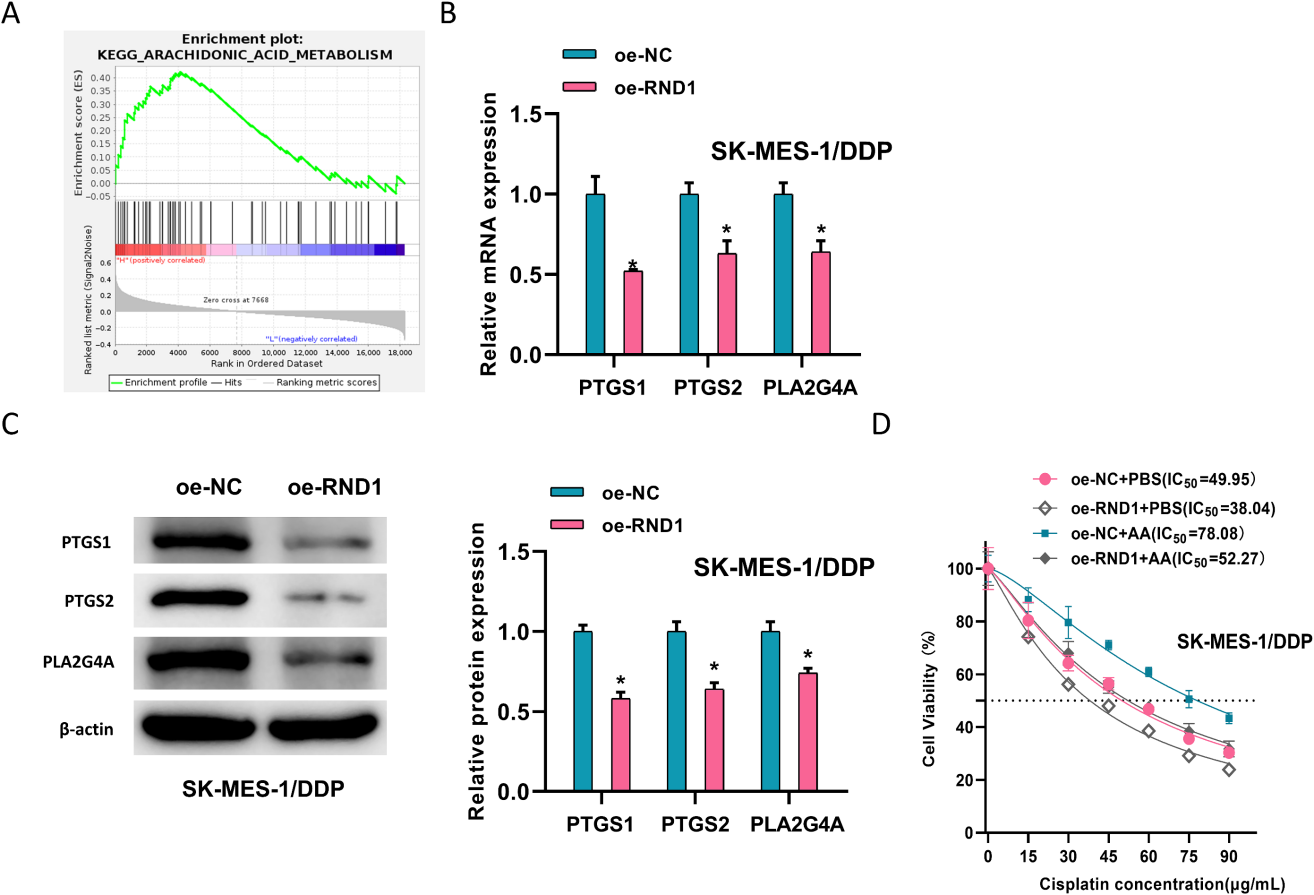
RND1 affects the AA pathway and regulates DDP resistance in LUSC. (A) GSEA results show enrichment of RND1 in the AA metabolism pathway. (B, C) qPCR and western blot analysis of mRNA and protein expression levels of AA metabolism‐related genes PTGS1, PTGS2, and PLA2G4A in drug‐resistant cells overexpressing RND1. (D) CCK8 analysis of IC_50_ values in different treatment groups (oe‐NC+PBS, oe‐RND1+PBS, oe‐NC+AA, and oe‐RND1+AA) after treatment of drug‐resistant cells. * represents *p* < 0.05.

This study hypothesized that RND1 was a tumor repressor gene in LUSC. To unveil the mechanism of RND1 in LUSC, based on the downloaded LUSC mRNA data from the TCGA database, RND1 was divided into a high‐expression group and a low‐expression group according to the median expression level of RND1. KEGG enrichment analysis was performed using GSEA software, and it was found that RND1 was significantly enriched in the AA metabolism pathway (Figure 2A). To investigate the relationship between the AA metabolism pathway and DDP resistance, SK‐MES‐1/DDP cells were transfected with oe‐NC and oe‐RND1. Expression of AA metabolism‐related proteins and mRNA was assessed by western blot and qPCR. Increased RND1 reduced the mRNA and protein expression of PTGS1, PTGS2, and PLA2G4A (Figure 2B,C). Based on this finding, we added AA to investigate whether RND1 exerts its drug resistance mechanism through this pathway. The CCK8 assay presented an IC_50_ value that significantly increased from 49.95 in the oe‐NC+PBS group to 78.08 in the oe‐NC+AA group. In the case of adding oe‐RND1 to PBS treatment, compared with the control group, the IC_50_ value significantly decreased, from 49.95 in the control group to 38.04. However, RND1 was also overexpressed during AA treatment, and the IC_50_ value was restored to the original level in the oe‐RND1+AA group (Figure 2D). This suggested that RND1 affected DDP resistance in LUSC by modulating AA metabolism.

### 3.3 FOXA2 Is an Upstream Regulator of RND1

**FIGURE 3:**
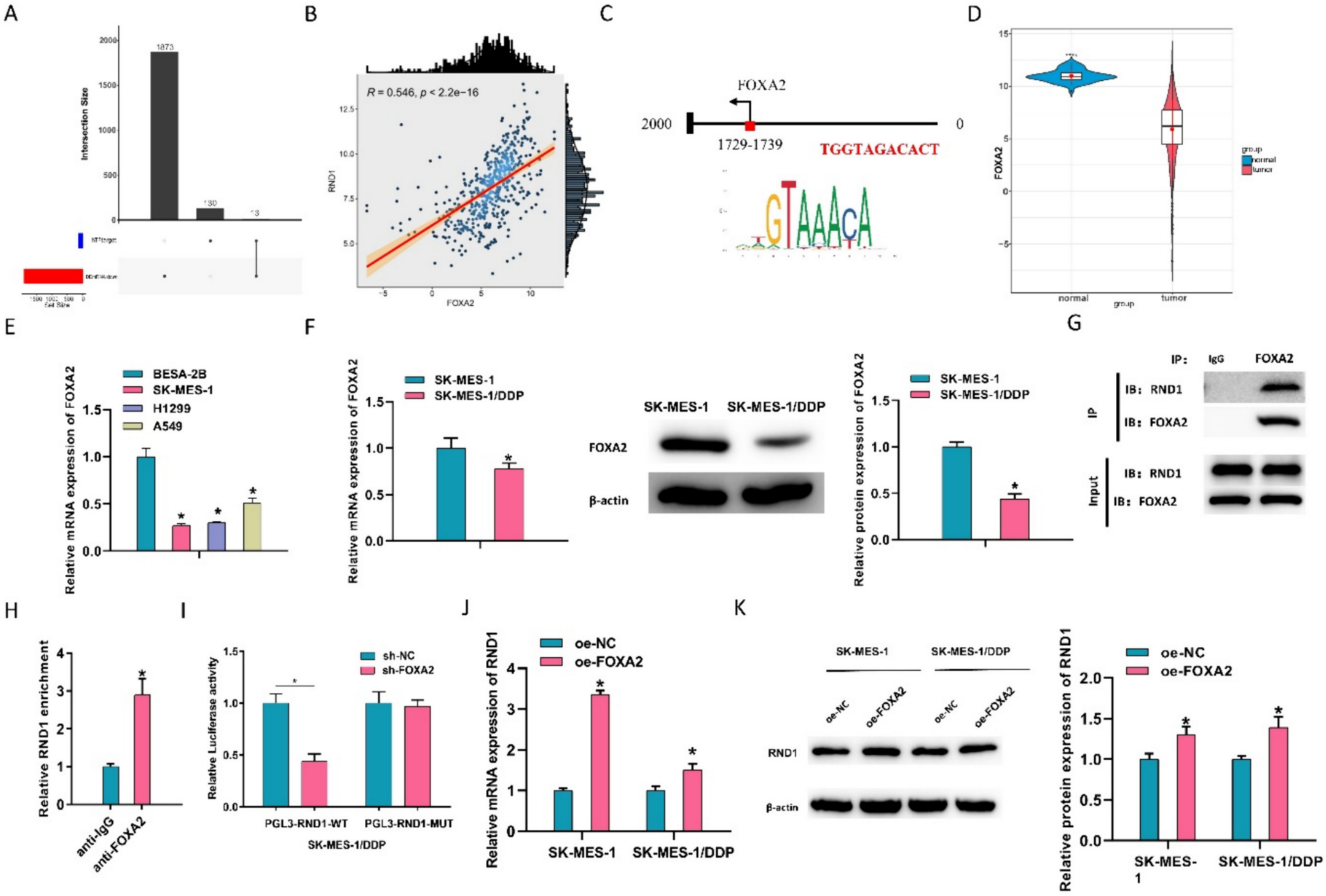
FOXA2 positively activates transcription of RND1. (A) hTFtarget predicted potential transcription factors upstream of RND1. (B) Pearson's analysis of RND1 and 13 potential transcription factors. (C) Motif prediction of the 2000‐bp region upstream of RND1 promoter using the JASPAR website. (D) Violin plot showing the expression levels of FOXA2 in normal and LUSC tissues by bioinformatics analysis. (E) qPCR detection of FOXA2 expression levels in human normal lung epithelial cells (BESA‐2B), LUSC cell line (SK‐MES‐1), and non–small cell lung cancer cell lines (H1299 and A549). (F) qPCR and western blot detection of FOXA2 mRNA and protein expression levels in DDP‐resistant and sensitive LUSC cell lines. (G) Co‐IP experiment of the relationship between protein of FOXA2 and protein of RND1. (H) CHIP experiment of the relationship between FOXA2 and RND1. (I) Dual‐luciferase assay of effect of FOXA2 knockdown on luciferase activity in PGL3‐RND1‐WT and PGL3‐RND1‐MUT treated groups. (J, K) qRT‐PCR and western blot detection of RND1 mRNA and protein expression levels after overexpression of FOXA2 in SK‐MES‐1/DDP and SK‐MES‐1 cells. * represents *p* < 0.05.

Increased RND1 suppressing DDP resistance in LUSC has been confirmed previously. To further comprehend the underlying mechanisms, this study further investigated upstream regulatory genes of RND1. The hTFtarget web tool was utilized to predict potential transcription factors upstream of RND1, and a total of 13 potential transcription factors were screened (Figure 3A). Pearson's analysis of RND1 with 13 potential transcription factors revealed the highest correlation between RND1 and FOXA2 (Figure 3B). Motifs were predicted in the 2000‐bp region upstream of the RND1 promoter (Figure 3C). The results of the *t*‐test also presented the downregulation of FOXA2 in LUSC (Figure 3D). Meanwhile, qPCR data unraveled that FOXA2 was downregulated in SK‐MES‐1, H1299, and A549 cells (Figure 3E).

To validate the prediction, CHIP and dual luciferase reporter gene assays were subsequently completed. Similar to the above experiment, FOXA2 expression in the DDP‐resistant SK‐MES‐1/DDP and the sensitive SK‐MES‐1 cell lines was first assayed by qPCR. FOXA2 mRNA was significantly downregulated in the resistant strain compared to the sensitive strain (Figure 3F). To further confirm this finding, a western blot was done on FOXA2 protein levels. FOXA2 protein level was also significantly downregulated in drug‐resistant strains (Figure 3F). The Co‐IP experiment results indicated an interaction between the FOXA2 protein and the RND1 protein (Figure 3G). CHIP presented that FOXA2 and RND1 could interact with each other (Figure 3H). A dual‐luciferase assay disclosed that the luciferase activity of cells expressing PGL3‐RND1‐WT significantly decreased when FOXA2 was silenced, while that of cells expressing PGL3‐RND1‐MUT showed no significant change (Figure 3I). RND1 expression was detected by qRT‐PCR after overexpression of FOXA2 in SK‐MES‐1/DDP and SK‐MES‐1 cells. The data illustrated a more significant increase in RND1 mRNA expression in DDP‐sensitive strains (Figure 3J). Meanwhile, the result was validated at the protein level. The expression level of RND1 protein increased in SK‐MES‐1/DDP and SK‐MES‐1 cells, and there was a more significant increase in RND1 protein expression in DDP‐sensitive strains (Figure 3K). These experiments indicated that FOXA2 was an upstream regulator of RND1, thus affecting the DDP resistance of cancer cells.

### 3.4 FOXA2 Activates RND1 to Regulate AA Metabolism and Repress DDP Resistance in LUSC

**FIGURE 4:**
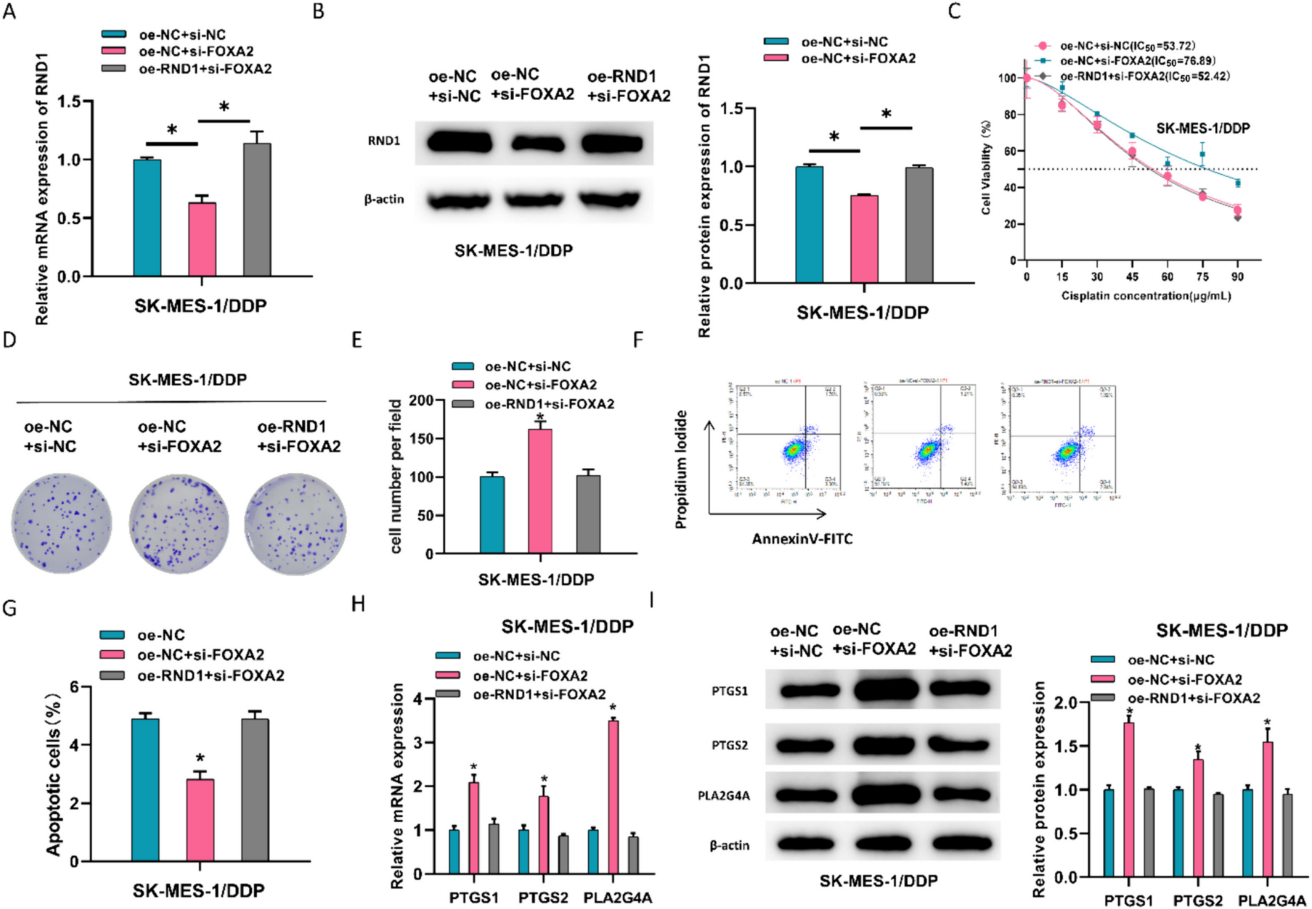
FOXA2 positively regulates RND1 to affect AA metabolism and inhibit DDP resistance in LUSC. (A) mRNA expression levels of RND1 in resistant cells were detected in different groups (oe‐NC+si‐NC, oe‐NC+si‐FOXA2, and oe‐RND1+si‐FOXA2). (B) Protein expression levels of RND1 in the above groups were detected by western blot. (C) IC_50_ values of the above groups were measured by CCK8 assay. (D, E) Colony formation experiments were performed to assess cell proliferation in the above groups, and images were taken and quantified. (F, G) Flow cytometry was used to measure cell apoptosis levels in different treatment groups, and a quantification graph was generated. (H, I) mRNA and protein levels of AA pathway‐related genes in different treatment groups were detected by qPCR and western blot, respectively. * represents *p* < 0.05.

In this experiment, we discovered the modulatory role of RND1 in the AA metabolism pathway and also validated the relationship between FOXA2 and RND1. To confirm the role of this functional axis, SK‐MES‐1/DDP cells were selected for experiments in different treatment groups, including oe‐NC+si‐NC, oe‐NC+si‐FOXA2 and oe‐RND1+si‐FOXA2. Firstly, we examined RND1 expression in each group. qRT‐PCR unveiled that FOXA2 knockdown led to decreased RND1 expression, while increased RND1 on this basis restored RND1 expression (Figure 4A). Similarly, western blot data displayed a similar trend, where the oe‐RND1+si‐FOXA2–treated group restored the decreased expression of RND1 caused by FOXA2 silencing (Figure 4B). CCK8 presented that with FOXA2 silencing, the IC_50_ value increased from 53.72 in the oe‐NC+si‐NC group to 76.89 in the oe‐NC+si‐FOXA2 group, while overexpression of RND1 on the basis of si‐FOXA2 could reduce the elevated IC_50_ value (Figure 4C). We treated drug‐resistant cells with IC_50_ and completed the colony formation assay. When FOXA2 was silenced, it drove cancer cell proliferation. However, when RND1 was overexpressed simultaneously, the cell proliferation rate lowered (Figure 4D,E). Flow cytometry analysis of apoptosis levels also exhibited a similar trend, where the oe‐NC+si‐FOXA2 treatment group hampered cell apoptosis, while overexpression of RND1 resulted in increased apoptosis levels (Figure 4F,G). Finally, the protein expression of PTGS1, PTGS2, and PLA2G4A related to AA metabolism was tested. When FOXA2 was silenced, levels of these genes were significantly increased, while they were partially restored when RND1 was overexpressed concurrently (Figure 4H,I). These findings suggested that FOXA2 activated RND1, thereby modulating AA metabolism to restrain DDP resistance in LUSC.

## 4 Discussion

This study demonstrated the influence of RND1 on DDP‐resistant cells in LUSC. RND1 was downregulated in LUSC. Overexpression of RND1 suppressed cancer cell proliferation. The association between RND1 and AA metabolism was verified, and the upstream transcription factor FOXA2 of RND1 was identified through bioinformatics methods. FOXA2 transcriptionally activated RND1. Both genes were downregulated in LUSC to activate the AA metabolism pathway and ultimately facilitate DDP resistance in LUSC.

RND1 is a small GTPase belonging to the RND subfamily. Like other Rho GTPases, the transcript of RND1 is aberrant in cancer. RND1 level is suppressed in highly invasive breast cancer, hepatocellular carcinoma, and high‐grade glioma, and it exhibits tumor‐repressive effects [20, 21, 24]. In contrast, RND1 is upregulated in low‐grade breast tumors, gastric cancer cell lines, and esophageal squamous cell carcinoma [25, 26, 27]. Hence, we did bioinformatics and experiments on LUSC tissues and cells and disclosed that RND1 was downregulated. We speculated that RND1 may exert a tumor‐repressive role in LUSC. In Section 3, the repression of increased RND1 on cancer cell progression was confirmed. Additionally, drug resistance is a common and challenging issue in LUSC therapy. Based on this, we speculated that aberrant RND1 expression may be linked to DDP resistance in LUSC. It is worth noting that current research on RND1 in cancer mainly focuses on phenotypic studies such as EMT [21, 28, 29], while research on drug resistance is relatively limited. Thus, this work fills the knowledge gap regarding the role of RND1 in LUSC drug resistance. How RND1 affects DDP resistance in LUSC was analyzed in depth. The data suggested that RND1 was associated with the AA metabolism pathway, and its upregulation constrained the synthesis and metabolism of AA. An essential component of lipid metabolism, AA is intimately linked to the onset and progression of malignancies [30]. Through manipulation of RND1, AA was downregulated, thus repressing the malignant progression of LUSC cells and increasing sensitivity to DDP.

The molecular mechanism of RND1 in cancer is currently less reported, and its abnormal expression may be manipulated via epigenetic mechanisms. Methylation of the RND1 promoter and deacetylation of histone hinder transcription of RND1 [31, 32]. Transcription factors may also affect RND1 expression through modulatory mechanisms. In breast cancer, Oct4 can bind to the RND1 promoter and restrain its transcription [33]. In LUSC, increased miR‐4652‐5p lowered RND1 expression and facilitated the malignant progression of cancer cells [29]. Therefore, we focused on upstream modulatory factors of RND1 and manifested that FOXA2 positively manipulated RND1 expression. Bioinformatics analysis and experimental validation confirmed that FOXA2 was lowly expressed in LUSC tissues and cells. FOXA2, a transcription factor, is a cell‐type–specific modulatory factor required for organ development [34]. In addition, FOXA2 participates in manipulating various cancers, including endometrial cancer [35], gastric cancer [36], and prostate cancer [37]. In this study, functional experiment results presented that upregulating FOXA2 or RND1 expression significantly hindered the DDP resistance of LUSC cells. Meanwhile, experimental evidence confirmed that AA was a crucial mediator, and reversal of DDP resistance caused by FOXA2 downregulation could be achieved by restoring AA levels.

The findings have certain theoretical and clinical significance. Firstly, the crucial role of the FOXA2‐RND1‐AA axis in DDP resistance in LUSC was revealed, expanding the understanding of the mechanism of drug resistance in LUSC. Furthermore, this axis became a novel target, providing a new approach to the treatment of drug‐resistant LUSC. By activating FOXA2, stabilizing RND1, and modulating AA levels, it may be possible to enhance the sensitivity of LUSC patients to DDP treatment and improve patient prognosis. This study does still have some limitations, though. The experimental data mainly rely on in vitro cell models, and validation is needed through in vivo models and clinical samples. Moreover, clarification is required on the mutual modulatory mechanisms of FOXA2, RND1, and AA in DDP resistance in LUSC. In conclusion, it is anticipated that these findings will offer fresh concepts and approaches for creating novel treatment plans and enhancing the effectiveness of existing ones for LUSC patients. Further research and clinical validation are needed to refine this mechanism and ultimately apply it to clinical practice.

## 5 Conclusion

In summary, this study investigated and validated that FOXA2 could transcriptionally activate RND1 by downregulating the signaling pathway of AA metabolism, thereby reducing the resistance of LUSC cells to DDP. This may provide a new theoretical basis for targeted research on improving the sensitivity of LUSC to DDP.

## Author Contributions

Conception and design: Yafu Zhou. Provision of study materials or patients: Huiguo Chen. Collection and assembly of data: Jianhua Yan and Shengying Xiao. Data analysis and interpretation: Qi Yao and You Peng. Manuscript writing: Chunchu Kong and Jinsong Yang. Final approval of manuscript: all authors.

## Ethics Statement

The authors have nothing to report.

## Consent

The authors have nothing to report.

## Conflict of Interests

The authors declare no conflicts of interest.

## Supporting information


**Appendix S1** Supporting Information

## Data Availability

The data that support the findings of this study are available from the corresponding author upon reasonable request.
